# Case report: Disseminated *Scedosporium apiospermum* infection with invasive right atrial mass in a heart transplant patient

**DOI:** 10.3389/fcvm.2022.1045353

**Published:** 2022-10-31

**Authors:** Baudouin Bourlond, Ana Cipriano, Julien Regamey, Matthaios Papadimitriou-Olivgeris, Christel Kamani, Danila Seidel, Frederic Lamoth, Olivier Muller, Patrick Yerly

**Affiliations:** ^1^Department of Cardiology, Lausanne University Hospital and University of Lausanne, Lausanne, Switzerland; ^2^Infectious Diseases Service, Department of Medicine, Lausanne University Hospital and University of Lausanne, Lausanne, Switzerland; ^3^Department of Nuclear Medicine and Molecular Imaging, Lausanne University Hospital and University of Lausanne, Lausanne, Switzerland; ^4^Department I of Internal Medicine, Faculty of Medicine, University Hospital Cologne, University of Cologne, Cologne, Germany; ^5^Center for Integrated Oncology Aachen Bonn Cologne Dusseldorf (CIO ABCD), University of Cologne, Cologne, Germany; ^6^Excellence Center for Medical Mycology (ECMM), University of Cologne, Cologne, Germany; ^7^Excellence Cluster on Cellular Stress Responses in Aging-Associated Diseases (CECAD), University of Cologne, Cologne, Germany; ^8^Department of Laboratories, Institute of Microbiology, Lausanne University Hospital and University of Lausanne, Lausanne, Switzerland

**Keywords:** *Scedosporium apiospermum*, *Lomentospora prolificans*, PET/CT, infective endocarditis, mycoses, heart tranplantation, heart failure

## Abstract

*Scedosporium apiospermum* associated endocarditis is extremely rare. We report a case of a disseminated *S. apiospermum* infection with an invasive right atrial mass in a 52-year-old male, 11 months after heart transplantation, referred to our institution for an endogenous endophthalmitis with a one-month history of diffuse myalgias and fatigue. The patient had been supported two times with extracorporeal membrane oxygenation (ECMO) during the first three postoperative months. The echocardiography on admission revealed a mass in the right atrium attached to a thickened lateral wall. The whole-body [^18^F]FDG PET/CT revealed systemic dissemination in the lungs, muscles, and subcutaneous tissue. Blood cultures were positive on day three for filamentous fungi later identified as *S. apiospermum*. The disease was refractory to a 3-week dual antifungal therapy with voriconazole and anidulafungin in addition to reduced immunosuppression, and palliative care was implemented.

## Introduction

Fungal infective endocarditis (FE) remains the most serious form of infective endocarditis, associated to a high mortality rate (∼50%) (1). In about half of the cases, *Candida* species account for FE, *Aspergillus* spp., account for one fourth of the cases (2). Non-*Aspergillus* molds infective endocarditis account for the remaining of cases, with *Scedosporium* spp. being seldom identified.

*Scedosporium* spp. are filamentous fungi, ubiquitous in the environment and commonly found in temperate climates. *Scedosporium apiospermum* and *Lomentospora prolificans* (formerly *S. prolificans*) (S/L) are the major pathogens in humans (3). Infection occurs after inhalation of spores into the lungs or paranasal sinuses or through direct skin inoculation after traumatic injuries or during surgery. Medical care advances increased the number of immunocompromised patients with non-*Aspergillus* mold infections (NAIMI). In solid organ transplant (SOT) recipients, approximately 25% of NAIMI are caused by S/L, representing 1% of all fungal infections in these patients (4, 5). In this population, pretransplant colonization of the respiratory tract (lung transplant recipients), prior amphotericin B treatment, and enhanced immunosuppression, as a treatment for organ rejection, represent the major associated risk factors for NAIMI (6).

Scedosporiosis and lomentosporiosis are of particular concern due to intrinsic resistance to most available antifungal drugs. Outcomes seem to be better with *S. apiospermum* infection, which presents a better response to antifungal agents. Voriconazole appears to have the best *in vitro* activity and is considered the drug of choice by most international guidelines (7). Combined antifungal therapy with echinocandins may be used against *S. apiospermum*, as a synergistic effect has been described (6, 8, 9). Where possible, surgical debridement and immunosuppression reduction should be part of the treatment management (6, 9).

## Case presentation

**FIGURE 1:**
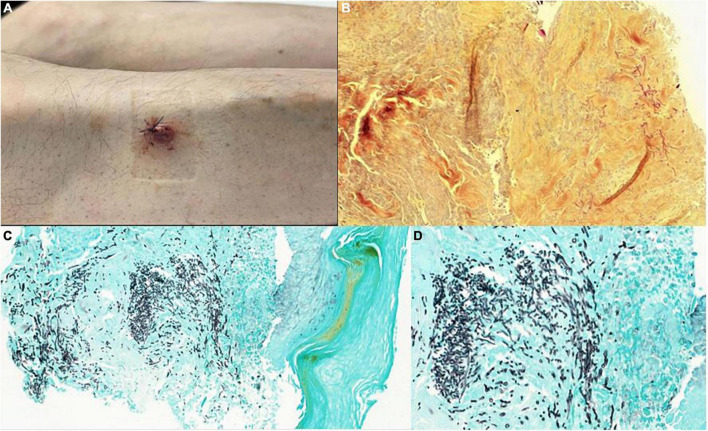
Painful purplish cutaneous nodules **(A)**, pathology examination with Periodic Acid Schiff coloration 10x **(B)**, and colorations with GROCOTT stain of cutaneous nodules showing regular septate filaments connected with 45-degree divisions 10x **(C)** and 40x **(D)**.

A 52-year-old male patient was referred to our hospital by the ophthalmologist for additional work-up in the context of endogenous endophthalmitis. He reported a one-month history of migratory myalgia without fever, one-week history of abdominal pain with vomiting, atraumatic eyes pain for two days, and an isolated cutaneous nodule on the right inferior limb (Figure 1, Panel A). He had received a heart transplantation for dilated cardiomyopathy 11 months prior to admission and had been on a left ventricular assist device (Heartmate 3TM) for three years before transplantation. His transplant operation had been complicated with primary graft failure necessitating eight days of mechanical support with veno-arterial ECMO. Moreover, he had suffered from a nosocomial SARS-CoV2 infection on the third post-operative month, with acute respiratory distress syndrome treated with tocilizumab, and refractory hypoxemia necessitating 18 days of venovenous ECMO. In both cases, peripheral cannulation of the right atrium was used.

At presentation (day 1), he was on immunosuppressive therapy with prednisolone 12.5 mg od, tacrolimus 3 mg bid, and mycophenolate mofetil 720 mg bid. On admission, blood pressure was 140/76 mmHg with a resting heart rate of 70 beats per minute and a temperature of 36.3°C. Physical examination was noteworthy for diffuse right eyelid edema, a painless purplish cutaneous nodule of the outer edge of the tibia (Figure 1, Panel A), and asymmetric erythema of the right ankle. Cardio-pulmonary, neurological and abdominal examinations were regular.

## Diagnosis, management and follow-up

**FIGURE 2:**
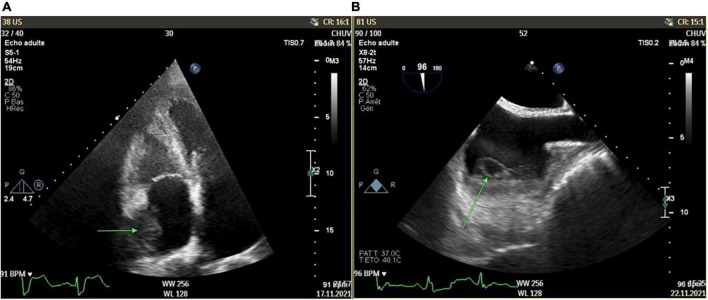
Right atrium mass (15 × 18 mm) with thickened lateral wall without associated tricuspid valve anomalies as shown in transthoracic echocardiography [green arrow, Panel **(A)**] and transesophageal echocardiography [green arrow, Panel **(B)**].

**FIGURE 3:**
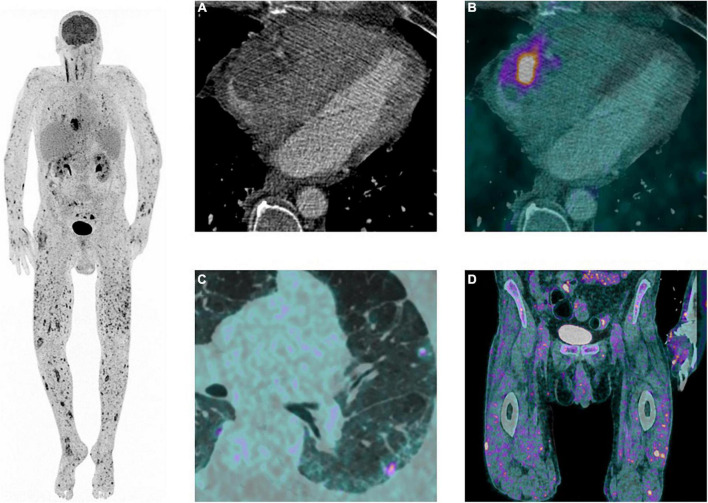
^18^F-fluodeoxyglucose ([^18^F]FDG) Positron emission tomography (PET)/computed tomography angiography (CTA) findings. Left: Maximum intensity projection image of the whole body showing pathological focal diffuse [^18^F]FDG uptake of the trunk as well as the upper and lower extremities. Right: **(A)** Cardiac CTA showing an heterogeneous mass with irregular margins, in contact with the lateral wall of the right atrium with extension in the pericardial space. **(B)** Transaxial view of [^18^F]FDG PET/CTA showing pathological [^18^F]FDG accumulation of the right atrial mass, suggestive of an active infectious process. **(C)** Transaxial view of [^18^F]FDG PET/CTA showing focal subpleural nodules with increase [^18^F]FDG accumulation in the left lung, suggestive of septic emboli. **(D)** Coronal view of [^18^F]FDG PET/CT showing focal increased cutaneous and subcutaneous [^18^F]FDG accumulation in the proximal part of the lower limbs, suggestive of septic emboli.

The cardiac workup included a transthoracic echocardiography revealing an atrial mass measuring 15 × 18 mm, with a thickened lateral wall without associated tricuspid valve anomalies (Figure 2A). These findings were confirmed with transoesophageal echocardiography, which showed an abscessed pattern in this mass and moving filaments (Figure 2B). The fundoscopy revealed bilateral endogenous endophthalmitis and Roth spots. Laboratory tests showed increased inflammatory parameters (CRP: 163 mg/L, procalcitonin: 0.66 mcg/L), with relatively low leucocyte count (4.8 G/L) and a KDIGO 1 acute kidney injury. Blood cultures were collected. In order to characterize the nature of the right atrial mass, a whole-body [^18^F]FDG PET/CT after 24 h of dietary preparation and heparin pre-administration to suppress the physiological myocardial FDG uptake was performed (10). It showed an intense FDG accumulation of the right atrial mass with extension to the epicardium, which was suggestive of an active infectious process. Septic emboli in the lungs, muscles, and subcutaneous tissue were also evident (Figure 3). Brain magnetic resonance imaging (MRI) also highlighted multiple brain lesions.

The first suspected diagnosis was bacterial endocarditis with secondary endogenous endophthalmitis (*Staphylococcus aureus*, streptococci, or enterococci as the most likely pathogens). Therefore, intravenous empirical antibiotic therapy with vancomycin and co-amoxicillin was started while awaiting blood cultures and the culture of a vitreous sample. Twenty-four hours later, due to increased serum beta-D-glucan antigen (>500 pg/ml) and negative blood cultures a fungal infection was suspected and liposomal amphotericin B (5 mg/kg) was initiated. The immunosuppression was reduced to the minimum with prednisone 5 mg and tacrolimus for trough levels at 5–7 ug/L. Blood cultures were positive for filamentous fungi on day 3. *S. apiospermum* was identified as the causative pathogen three days later (day 6) and liposomal amphotericin B (minimum inhibitory concentration, – MIC 2.0 mg/l) switched to combination therapy with voriconazole (6 mg/kg bid the first day and then 4 mg/kg bid; MIC 0.25 mg/l) and anidulafungin (200 mg the first day and then 100 mg/day; MIC 1 mg/l). The endophthalmitis was treated with an intravitreal injection of ceftazidime and vancomycin. After *S. apiospermum* was identified, two intravitreal injections of voriconazole 0.5 mg were performed. Surgical resection of the right atrial mass was early discussed but refrained considering the invasion of the atrial wall, the disseminated disease, and the patient’s poor general condition with worsening asthenia and daily loss of weight despite enteral and then parenteral nutrition.

Despite dual antifungal therapy and reduced immunosuppression, the patient presented with ongoing fever, declining general condition, and a confusional state favored by brain abscesses and severely impaired vision. During this time, cardiac function remained stable, and no local complications of the atrial mass such as atrial obstruction, wall rupture with pericardial effusion, or invasion of the tricuspid valve were observed during serial echocardiographic assessments. Nevertheless, he developed multiple painful purplish cutaneous nodules, two of whom were biopsied and revealed regular septate filaments connected with 45-degree divisions on the Periodic Acid Schiff (PAS) and GROCOTT coloration (Figure 1). These lesions were extremely painful, and pain management was a true challenge in the context of severe renal impairment and worsening liver function related to the toxicity of antifungal therapies. A high dose of intravenous fentanyl was necessary in addition to anxiolytic treatment.

After three weeks of optimal medical treatment, cerebral MRI and [^18^F]FDG PET/CT were performed and showed no regression of the multiple lesions. The clinical course continued to be unfavorable, with the patient’s general condition declining rapidly. At his request, palliative care was started. He was transferred to a palliative institution, where he deceased a few days later.

## Discussion/Conclusion

Scedosporiosis/lomentosporiosis, even though rare, is of particular concern due to intrinsic resistance to available antifungal drugs. Current clinical guidelines recommend voriconazole alone or in combination and surgical source control (7, 8).

By microscopy, the morphological characteristics of these fungi are well characterized and distinct with the presence of ovoid or pyriform conidia arising as a single terminal spore from a long tiny conidiophore (*S. apiospermum* complex) or in clusters from a short flask shaped conidiophore (*L. prolificans*).

Invasive scedosporiosis represents 13–33% of infections due to non-*Aspergillus* molds (11). Fifty-seven cases of scedosporiosi*s* were described in the United States, while only 5 cases have been reported with heart involvement worldwide (12), as shown in Table 1. In these cases, despite antifungal treatments (itraconazole, voriconazole, amphotericin B), there was no survivor (6, 13).

**TABLE 1:** Literature review of infective endocarditis caused by *Scedosporium* spp. in solid transplant patients.

Case	Reference	Sex(M/F)/ age(years)	Organ transplanted	Year of transplant	Year of infection	Immunosuppression	Insolation site	Disseminated infection	Cardiac site	Treatment	Cardiac surgery	Outcome
1	(18)	F/37	Lung	2008	2008	T, M, P	Blood, bronchial secretion, cutaneous biopsy	Yes	Native mitral valve	Voriconazole, caspofungin, terbinafine	Yes	Deceased
2	(19)	M/35	Lungs and liver	2012	2012	B, T, M, P	Autopsy	Yes	No precision	Caspofungin, voriconazole	No	Deceased
3	(20)	M/19	Heart	2013	2013	B, T, M, P	Blood	Yes	Papillary muscle of both ventricles	Amphotericin-B	No	Deceased
4	(21)	M/70	Heart	2011	2014	T	Blood, valve culture, synovial fluid	Yes	Native tricuspid valve	Posaconazole, terbinafine	Yes	Deceased
5	(22)	M/62	Lung	2014	2014	Unknown	Bronchial aspirate, surgical incision, post-mortem analysis	Yes	Left ventricle	Amphotericin-B	No	Deceased
6	Present study	M/52	Heart	2020	2021	T, M, P	Blood	Yes	Right atrium	Liposomal amphotericin B, voriconazole, anidulafungin	No	Deceased

B: basiliximab for induction, M: mycophenolate mophetil, P: prednisone, T: tacrolimus.

Our patient was on tacrolimus, which was previously shown to have synergistic effect with azoles *in vitro* and in a *Galleria mellonella* larvae model (14). However, this synergistic effect is obtained at high concentrations of tacrolimus (i.e., beyond the therapeutic range) and is counterbalanced by the strong immunosuppressive effect of this calcineurin inhibitor which may favor invasive fungal infections. As previously shown, despite this synergistic effect, the outcome was unfavorable for our patient.

It is noteworthy that novel antifungal agents, such as fosmanogepix (inhibitor of the glycosylphosphatidylinositol biosynthesis) and olorofim (inhibitor of the dihydroorotate dehydrogenase), which are currently in phase II-III clinical trials, demonstrated potent *in vitro* activity and *in vivo* efficacy in murine models against *S. apiospermum* and *L. prolificans*, and therefore represent promising therapies for scedosporiosis/lomentosporosis in the future (9, 15, 16). Our case illustrates a unique presentation of this rare infection with an intra-cardiac mass in the right atrium infiltrating its lateral wall and the tricuspid annulus, with disseminated disease (bilateral endophthalmitis, lungs, brain, muscles, and subcutaneous tissue). The patient was supported with ECMO twice following heart transplantation, which suggests that the infection may have spread from the venous cannula of the ECMO in the right atrium. Moreover, in addition to his post-transplant immunosuppression, he received tocilizumab to treat his severe SARS-COV-2 infection. This IL-2 receptor inhibitor has been associated with an increased risk for fungal infections (17). This kind of infection can happen in transplant patients, especially if they previously had primary graft dysfunction (PGD) or veno-arterial ECMO.

Our case highlights the importance of early detection of the pathogen by fungal culture or indirect marker such as the beta-glucan test in serum (13). In addition to surgery and reduction of immunosuppression when possible, early identification and treatment may improve the chance of recovery and survival.

## Data availability statement

The original contributions presented in the study are included in the article/supplementary material, further inquiries can be directed to the corresponding author.

## Ethics statement

Written informed consent was obtained from the individual(s) for the publication of any potentially identifiable images or data included in this article.

## Author contributions

All authors approved the manuscript, vouch for the accuracy, and completeness of the data.
