# Erratum: Disseminated Scedosporium apiospermum infection with invasive right atrial mass in a heart transplant patient

**DOI:** 10.3389/fcvm.2023.1173503

**Published:** 2023-03-08

**Authors:** Frontiers Production Office

**Affiliations:** Frontiers Media SA, Lausanne, Switzerland

**Keywords:** Scedosporium apiospermum, Lomentospora prolificans, PET/CT, infective endocarditis, mycoses, heart tranplantation, heart failure

An Erratum on Disseminated Scedosporium apiospermum infection with invasive right atrial mass in a heart transplant patient By Bourlond B, Cipriano A, Regamey J, Papadimitriou-Olivgeris M, Kamani C, Seidel D, Lamoth F, Muller O and Yerly P. (2022) Front. Cardiovasc. Med. 9:1045353. doi: 10.3389/fcvm.2022.1045353

An omission to the funding section of the original article was made in error. The following sentence has been added: “Open access funding was provided by the University of Lausanne.”

The original version of this article has been updated.

